# ABCB1 SNP predicts outcome in patients with acute myeloid leukemia treated with Gemtuzumab ozogamicin: a report from Children’s Oncology Group AAML0531 Trial

**DOI:** 10.1038/s41408-019-0211-y

**Published:** 2019-05-21

**Authors:** Roya Rafiee, Lata Chauhan, Todd A. Alonzo, Yi-Cheng Wang, Ahlam Elmasry, Michael R. Loken, Jessica Pollard, Richard Aplenc, Susana Raimondi, Betsy A. Hirsch, Irwin D. Bernstein, Alan S. Gamis, Soheil Meshinchi, Jatinder K. Lamba

**Affiliations:** 1 Department of Pharmacotherapy and Translational Research, Center for Pharmacogenomics, College of Pharmacy, University of Florida, Gainesville, FL USA; 2 Keck School of Medicine, University of Southern California, Los Angeles, CA USA; 3 Children’s Oncology Group, Monrovia, CA USA; 4 Mansoura University, Mansoura, Egypt; 5 Hematologics Inc, Seattle, USA; 6 Dana-Farber/Boston Children’s Cancer Center and Blood Disorders Center, Boston, MA USA; 7 Harvard Medical School, Boston, MA USA; 8 Division of Oncology, Children’s Hospital of Philadelphia, Philadelphia, PA USA; 9 Department of Pathology, St. Jude Children’s Hospital, Memphis, TN USA; 10 Children’s Hospitals and Clinic of Minnesota, University of Minnesota, Minneapolis, MN USA; 11 Clinical Research Division, Fred Hutchinson Cancer Research Center, Seattle, WA USA; 12 Division of Hematology/Oncology/Bone Marrow Transplantation, Children’s Mercy Hospitals and Clinics, Kansas City, MO USA

**Keywords:** Genomics, Genetics research

## Abstract

Gemtuzumab-ozogamicin (GO), a humanized-anti-CD33 antibody linked with the toxin-calicheamicin-γ is a reemerging and promising drug for AML. Calicheamicin a key element of GO, induces DNA-damage and cell-death once the linked CD33-antibody facilitates its uptake. Calicheamicin efflux by the drug-transporter PgP-1 have been implicated in GO response thus in this study, we evaluated impact of *ABCB1*-SNPs on GO response. Genomic-DNA samples from 942 patients randomized to receive standard therapy with or without addition of GO (COG-AAML0531) were genotyped for ABCB1-SNPs. Our most interesting results show that for rs1045642, patients with minor-T-allele (CT/TT) had better outcome as compared to patients with CC genotype in GO-arm (Event-free survival-EFS: *p* = 0.022; and risk of relapse-RR, *p* = 0.007). In contrast, no difference between genotypes was observed for any of the clinical endpoints within No-GO arm (all *p* > 0.05). Consistent results were obtained when genotype groups were compared by GO and No-GO arms. The in vitro evaluation using HL60-cells further demonstrated consistent impact of rs1045642-T-allele on calicheamicin induced DNA-damage and cell-viability. Our results show the significance of *ABCB1* SNPs on GO response in AML and warrants the need to investigate this in other cohorts. Once validated, *ABCB1*-SNPs in conjunction with CD33-SNPs can open up opportunities to personalize GO-therapy.

## Introduction

Gemtuzumab ozogamicin (GO), a CD33-antibody conjugated to cytotoxin-calicheamcin^1^ is a re-emerging and promising drug in AML treatment. In light of encouraging results from several randomized studies^2–8^, recent re-approval of GO by FDA is a big step in treatment of AML. Given the anticipated increase in GO use, there is an urgent unmet need to identify biomarkers that can improve our ability to personalize GO. We recently reported a splicing-polymorphism in CD33, with significant impact on GO response^9^, these encouraging results prompted us to investigate additional biomarkers that can impact GO-response.

Since anti-leukemic effects of GO are due to calicheamicin-induced DNA damage, intracellular abundance of calicheamicin is critical for its anti-leukemic effect. PgP1, encoded by *ABCB1*, influences the accumulation of calicheamicin and its expression levels has been associated with in vitro GO-sensitivity and clinical response in AML patients^10–14^. In vitro cellular sensitivity of unconjugated-calicheamicin varies >100,000-fold between the most-resistant and most-sensitive AML^15^. Unfortunately, the impact of genetic-polymorphisms in *ABCB1* on the clinical-efficacy of GO has never been investigated. In this study, we evaluated *ABCB1* SNPs in pediatric AML patients randomized to the addition of two doses of GO to standard chemotherapy^8^.

## Materials and methods

### Patients and treatment

This study included specimens from pediatric de novo AML patients (ages 0–29 years) randomized to receive standard five-course chemotherapy (No-GO arm, *n* = 511) or the same chemotherapy with addition of two doses of 3 mg/m^2^ GO (GO arm, *n* = 511) during induction I and intensification II in COG-AAML0531 trial. The study design, treatment regimen and clinical outcome details have been reported previously^8^. Low-risk group included presence of t(8;21), inv(16), or t(16;16). High-risk group included monosomy 7, monosomy 5/5q deletion, or persistent disease at end of induction 1 and all high-risk group patients received allogeneic stem cell transplant. Patients with absence of low or high-risk features were assigned intermediate-risk group and received HSCT if suitable donor was available^8,16^. Specimens from patients who consented for biology studies were used in this study. The institutional review boards of all participating institutions approved the clinical protocol, and the COG-Myeloid Disease Biology Committee approved this research.

### Genotyping

In this study, we genotyped 12 *ABCB1* SNPs in genomic DNA from 942 children and young adults enrolled on COG-AAML0531. Genotyping was performed using the Sequenome platform at the Biomedical Genomics Center, University of Minnesota. Three SNPs with minimum allele frequency of <0.1 or call-rate of <0.95 were excluded (Supplementary Table 1). Of the 9 SNPs that were included in the study 3 SNPs (rs1045642; rs1128503; and rs2032582) occur in partial LD with each other.

### Statistical analysis

Statistical analysis was performed to compare clinical endpoints: overall-survival (OS), and event-free-survival (EFS) from study entry, disease-free-survival (DFS) from end of Course 1 (EOC1) and risk of relapse (RR) from EOC1. Analysis was performed: (i) between genotype groups within each arm and (ii) between GO and No-GO arms within different genotype groups in all patients as well as within risk-groups^8^ using Cox regression and Fisher’s exact test.

### In vitro evaluation of ABCB1 SNP-rs1045642

HL-60 cells, a promyelocytic cell line with low näive expression of ABCB1 (ATCC, Manassas, VA, USA) was select to evaluate ABCB1 SNP -rs1045642. Cell line was cultured in medium containing 10% FBS and 1% Glutamine (Invitrogen, USA) at 37 °C in humidified incubator containing 5% CO_2_ as per manufacturer’s instructions.

Cells were transfected with either control -pCl-neo or ABCB1 expression vectors: ABCB1- rs1045642-C, and rs1045642-T using nucleofector2b (Lonza, Switzerland) as per manufacture’s protocol. Forty-eight hours post-transfection cell surface expression levels of Pgp-1 was analyzed using ABCB1 mAB-UIC2 conjugated with Phycoerythrin (PE), (Abcam, ab93590) by BDTM FACS LSR II. 48 h post-transfection cells were exposed to 40 nM calicheamicin for 24 h and cell viability evaluated using NucRed Live (Invitrogen, USA) and AO-PI (Acridine orange and propidium iodide) staining.

We also evaluated calicheamicin induced DNA-damage in cells transfected with control and ABCB1-expression vectors using comet assays. Post-transfection, cells were treated with 40 nM calicheamicin for 45 min followed by immobilization in a bed of low (1%) melting agarose. Cells were treated with alkali to unwind the DNA. Electrophoresis and subsequent staining with SYBER gold nucleic acid gel stain (Invitrogen, USA) was performed and percent of DNA tail-length was estimated using fluorescence microscopy and image J software.

## Results

### Clinical response within GO and No-GO arms by ABCB1 SNP genotypes

**Fig. 1:**
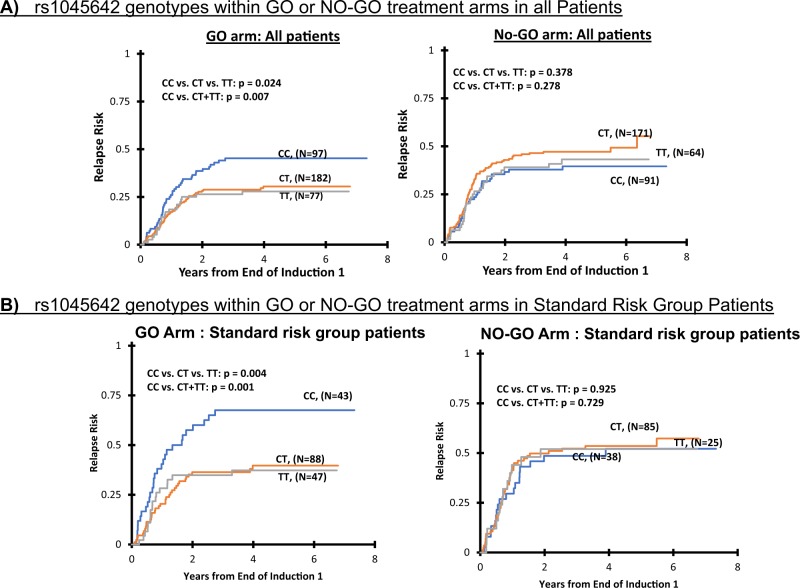
Association of *ABCB1* SNP rs1045642 with clinical outcome in patients from AAML0531 trial. Comparison of risk of relapse from end of course 1 by rs1045642 genotype within GO or No-GO arms in all patients (**a**) and within standard risk group patients (**b**)

Initial analysis evaluated association of *ABCB1* SNPs with clinical outcome within GO arm or No-GO arm. rs1045642 C>T, which is one of the most studied SNP within *ABCB1* showed significant association of genotype with response. Patients with minor allele (CT/TT) had a significantly better outcome with GO as compared to patients with CC genotype (GO arm: 5 years EFS: CC = 44 ± 9%, CT = 55 ± 7%, TT = 56 ± 10%, CC vs. CT/TT *p* = 0.022; DFS, CC = 51 ± 10%, CT = 62 ± 7%, TT = 64 ± 11%, CC vs. CT/TT *p* = 0.044 and RR, CC = 45 ± 10%, CT = 30 ± 7% TT = 28 ± 10%, CC vs. CT/TT *p* = 0.007, Table 1; Fig. 1a). In contrast, no difference between genotypes was observed for any of the clinical endpoints within standard No-GO arm (all *p* > 0.05). Given that risk-groups impact clinical responses to GO and allele-frequency of rs1045642 SNP differ by risk-group classification (Supplementary Table 2), we evaluated *ABCB1* SNPs within low, standard and high-risk group patients by treatment arms. As shown in Fig. 1b, we observed significant association of rs1045642 SNP with outcome in GO arm within only standard-risk group patients (GO arm: EFS, *p* = 0.014, DFS, *p* = 0.006 and RR, *p* = 0.004 for CC vs. CT vs. TT genotypes, Fig. 1b; Supplementary Table 3).

**Table 1:** ABCB1 SNPs demonstrate significant association with clinical outcome in patients enrolled in GO arm of the randomized AAML0531 clinical trial

ABCB1 SNPs	Differences by ABCB1 genotypes within No-GO or GO arm									
ABCB1 SNP_rs1045642	No-GO ARM (rs1045642)					GO-ARM (rs1045642)				
	% ± 2SE%	% ± 2SE%	% ± 2SE%	*p*	*p*	% ± 2SE%	% ± 2SE%	% ± 2SE%	*p*	*p*
	CC (*N* = 132)	CT (*N* = 244)	TT (*N* = 93)	CC vs. CT vs. TT	CC vs. CT+TT	CC (*N* = 128)	CT (*N* = 238)	TT (*N* = 102)	CC vs. CT vs. TT	CC vs. CT+TT
5 years OS	63 ± 9%	62 ± 6%	70 ± 10%	0.323	0.371	59 ± 9%	66 ± 7%	71 ± 10%	0.123	0.068
5 years EFS	50 ± 9%	44 ± 6%	48 ± 11%	0.633	0.466	44 ± 9%	55 ± 7%	56 ± 10%	0.073	**0.022**
	CC (*N* = 91)	CT (*N* = 171)	TT (*N* = 64)	CC vs. CT vs. TT	CC vs. CT+TT	CC (*N* = 97)	CT (*N* = 182)	TT (*N* = 77)	CC vs. CT vs. TT	CC vs. CT+TT
5 years DFS from EOC1	56 ± 11%	50 ± 8%	55 ± 13%	0.441	0.472	51 ± 10%	62 ± 7%	64 ± 11%	0.126	**0.044**
5 years RR from EOC1	40 ± 11%	47 ± 8%	43 ± 13%	0.378	0.278	45 ± 10%	30 ± 7%	28 ± 10%	**0.024**	**0.007**

ABCB1 SNP_rs2235015	No-GO ARM (rs2235015)					GO ARM (rs2235015)				
	% ± 2SE%	% ± 2SE%	% ± 2SE%	*p*	*p*	% ± 2SE%	% ± 2SE%	% ± 2SE%	*p*	*p*
	GG (*N* = 300)	GT (*N* = 141)	TT (*N* = 24)	GG vs. GT vs. TT	GG+GT vs. TT	GG (*N* = 295)	GT (*N* = 141)	TT (*N* = 27)	GG vs. GT vs. TT	GG+GT vs. TT
5 years OS	64 ± 6%	63 ± 8%	66 ± 20%	0.848	0.954	65 ± 6%	68 ± 8%	54 ± 20%	0.450	0.247
5 years EFS	46 ± 6%	49 ± 9%	41 ± 20%	0.892	0.644	52 ± 6%	55 ± 8%	37 ± 19%	0.303	0.156
	GG (*N* = 212)	GT (*N* = 94)	TT (*N* = 16)	GG vs. GT vs. TT	GG+GT vs. TT	GG (*N* = 219)	GT (*N* = 111)	TT (*N* = 22)	GG vs. GT vs. TT	GG+GT vs. TT
5 years DFS from EOC1	52 ± 7%	55 ± 10%	49 ± 26%	0.934	0.719	60 ± 7%	60 ± 9%	41 ± 21%	0.286	0.118
5 years RR from EOC1	46 ± 7%	40 ± 10%	45 ± 27%	0.851	0.929	33 ± 7%	30 ± 9%	59 ± 22%	**0.046**	**0.016**

The bold values indicate statistically significant *p* value of <0.05

For a less frequent intronic-SNP rs2235015-G>T that occurs with the allele frequency of (0.207) presence of G allele (GG/GT genotypes) was associated with lower RR (GG/GT vs. TT, *p* = 0.016) only in GO arm, whereas no association between genotypes and outcome was observed in No-GO arm (*p* > 0.05, Table 1).

### Clinical response within ABCB1 SNP genotypes by GO and No-GO arms

**Fig. 2:**
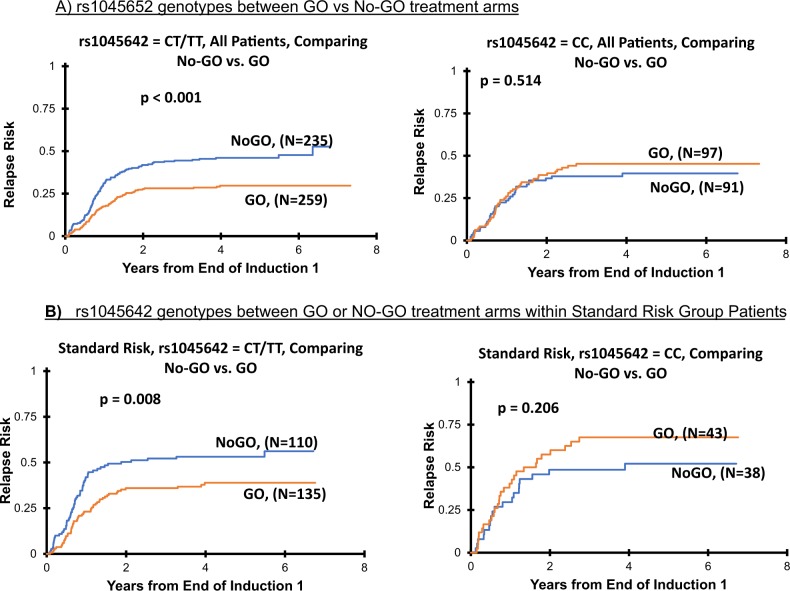
Association of *ABCB1* SNP rs1045642 with clinical outcome in patients from AAML0531 trial. Risk of relapse from end of course 1 comparison between GO and No-GO arms in all patients (**a**) or within standard risk group (**b**) within CC or CT/TT genotypes for rs1045642

Second analysis evaluated difference in clinical outcome comparing No-GO and GO treatment arms within different genotype groups for *ABCB1* SNPs and showed consistent with results as observed in within arm analysis above. Patients with rs1045642-CT/TT genotype had better EFS (No-GO vs. GO: 45 ± 5% vs. 55 ± 6%, *p* = 0.005), DFS (No-GO vs. GO: 51 ± 5% vs. 62 ± 6%, *p* = 0.008) and RR (No-GO vs. GO: 46 ± 7% vs. 30 ± 6%, *p* < 0.001; Fig. 2; Table 2) when treated on GO containing arm as compared to No-GO-arm. In contrast no difference in outcome between GO vs. No-GO arms was observed for patients within rs1045642-CC genotype group (all *p* > 0.05).

**Table 2:** ABCB1 genotype demonstrates significant difference in clinical outcome in GO vs. No-GO arms in patients enrolled in randomized AAML0531 clinical trial

ABCB1 SNPs	Differences in No-GO vs GO treatment arms by ABCB1 SNP genotypes					
ABCB1 SNP_rs1045642	CC (*N* = 260)			TT/CT (*N* = 677)		
	%±2SE%	%±2SE%	*p*	%±2SE%	%±2SE%	*p*
	No-GO (*N* = 132)	GO (*N* = 128)		No-GO (*N* = 337)	GO (*N* = 340)	
5 years OS	63 ± 9%	59 ± 9%	0.851	64 ± 5%	67 ± 5%	0.391
5 years EFS	50 ± 9%	44 ± 9%	0.425	45 ± 5%	55 ± 6%	**0.005**
	No-GO (*N* = 91)	GO (*N* = 97)		No-GO (*N* = 235)	GO (*N* = 259)	
5 years DFS from EOC1	56 ± 11%	51 ± 10%	0.561	51 ± 7%	62 ± 6%	**0.008**
5 years RR from EOC1	40 ± 11%	45 ± 10%	0.514	46 ± 7%	30 ± 6%	**<0.001**

AB CB1 SNP_rs2235015	TT (*N* = 51)			GG/GT (*N* = 877)		
	%±2SE%	%±2SE%	*p*	%±2SE%	%±2SE%	*p*
	No-GO (*N* = 24)	GO (*N* = 27)		No-GO (*N* = 441)	GO (*N* = 436)	
5 years OS	66 ± 20%	54 ± 20%	0.579	64 ± 5%	66 ± 5%	0.449
5 years EFS	41 ± 20%	37 ± 19%	0.940	47 ± 5%	53 ± 5%	**0.049**
	No-GO (*N* = 16)	GO (*N* = 22)		No-GO (*N* = 306)	GO (*N* = 330)	
5 years DFS from EOC1	49 ± 26%	41 ± 21%	0.903	53 ± 6%	60 ± 6%	**0.036**
5 years RR from EOC1	45 ± 27%	59 ± 22%	0.585	44 ± 6%	32 ± 5%	**0.001**

*EOC1* end of course 1

The bold values indicate statistically significant *p* value of <0.05

This association of improved clinical outcome within patients with at least one T allele in response to GO addition was consistent across the three risk group categories. Within standard-risk group patients, presence of rs1045642-T allele was associated with improved outcome in GO recipients only (No-GO vs. GO; EFS: 37 ± 7% vs. 49 ± 8%, *p* = 0.006, DFS: 45 ± 10% vs. 57 ± 9%, *p* = 0.024 and RR: 53 ± 10% vs. 39 ± 9%, *p* = 0.008, Supplementary Table 4; Fig. 2b). Within low and high-risk group presence of T allele (CT/TT) was associated with lower RR (No-GO vs. GO; low-risk group RR: 35 ± 10% vs. 16 ± 8%, *p* = 0.005 and high-risk group RR: 64 ± 21% vs. 28 ± 18%, *p* = 0. 018) (Supplementary Table 4). In contrast for rs1045642-CC genotype, no difference in outcome within risk groups was observed in GO vs. No-GO arms (all *p* > 0.05). These results were consistent within genotype groups for the SNPs rs1128503 and rs2032582, which occurs in partial LD with rs1045642 (Supplementary Table 5) as well as for the intronic SNP-rs2235015 (Table 2).

A multivariate Cox regression analysis that included rs1045642, risk status, and CD33 expression (CD33 expression has been shown to impact GO response^16,17^) demonstrated that treatment-arm is significant predictor of outcome for rs1045642 CT/TT genotype (Hazard Ratio (HR) = 0.543 and *p* < 0.001 for DFS; HR = 0.428 and *p* < 0.001 for RR) similar results were observed for the rs2235015 GG/GT genotype (HR = 0.66 and *p* = 0.004 for DFS; HR = 0.56 and *p* < 0.001 for RR; Supplementary Table 6).

### Interaction between ABCB1 rs1045642 and CD33 splicing SNP rs12459419 on clinical outcome

We recently reported a splicing SNP-rs12459419 C>T in *CD33*, that results in loss of IgV domain (which is recognized by GO) to be significant predictor of clinical outcome in response to GO^9^. For this splicing SNP-CC genotype had significant benefit with almost 50% reduction of risk of relapse in GO arm as compared to standard No-GO arm, whereas no benefit of adding GO is seen in CT/TT genotype. In light of these results. we evaluated *ABCB1*-rs1045642 and *CD33*-rs12459419 SNP-SNP interaction within COG-AAML0531-GO arm. Within patients with *CD33* rs12459419-CC genotype, *ABCB1* SNP was not associated with outcome (GO arm, RR, rs1045642 CC = 35 ± 13.9%, CT = 28 ± 9.2%, TT = 22.6 ± 15.3%, *p* = 0.594), however in *CD33*-rs12459419-CT genotype group, presence of *ABCB1*-rs1045642-CC was associated with inferior outcome as compared to *ABCB1*-rs1045642 CT/TT genotypes (within rs12459419-CT RR: rs1045642- CC = 59.7 ± 16.3%, CT = 30.8 ± 11.4%, TT = 28.1 ± 15.4%, CC vs. CT vs. TT, *p* = 0.005, CC vs. CT/TT *p* = 0.001; DFS within rs12459419 CT: rs1045642- CC = 35.2 ± 15.5% CT = 65 ± 11.6% TT = 62.4 ± 16.7%, CC vs. CT vs. TT, *p* = 0.005, CC vs. CT/TT, *p* = 0.001).

### In vitro evaluation of ABCB1 rs10456C>T SNP on calicheamicin response

**Fig. 3:**
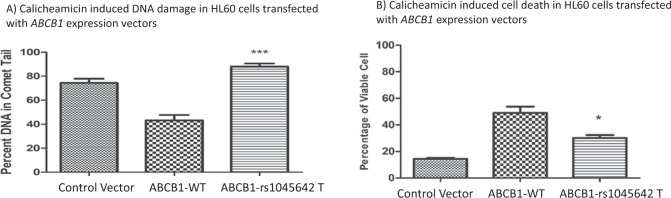
**a** Comet Assay: percent tail DNA measured in HL60 cells transfected with ABCB1 expression constructs in response to calicheamicin treatment. Histograms represented fluoresce microscopy data were analyzed by Image J software to calculate average of tails per each group (***p < 0.001). **b** Calicheamicin reduced cell viability in HL-60 cells expressing ABCB1-1045642T as compared to ABCB1-WT as determined by AOPI. Dual-fluorescence viability, Acridine orange (AO) and propidium iodide (PI) (*p < 0.05)

For in vitro evaluation, we selected HL-60 cell line with low naive expression of ABCB1. Cells were transfected with empty expression vector or expression vectors expressing ABCB1 WT (rs1045642 C) or ABCB1 -rs1045642 T variant. Cell surface Pgp-1 was determined 48 h post-transfection using UIC2 ABCB1 antibody conjugated with PE. The mean fluorescence intensity (MFI) analyzed on a 5 decade log scale (1–100,000) confirmed over expression in transfected cells compare to naive cells. DNA damage was evaluated using comet assay that demonstrated significant increased in DNA damage in response to calicheamicin treatment (40 nM for 0.75 h) in HL60 expressing ABCB1-rs1045642T variant allele as compared to ABCB1-WT (*p* < 0.05; Fig. 3a). This implies reduced accumulation of calicheamicin as a potential reason for reduced DNA damage in cells expressing ABCB1-WT. Corresponding to this calicheamicin induced cell death was greater in cells expressing ABCB1-C3435T as compared to the ABCB1-WT (Fig. 3b, *p* < 0.05) expression constructs when tested using NucRed live and AOPI staining.

## Discussion

*ABCB1* has been previously shown to impact response to gemtuzumab ozogamicin. In this study, we evaluated whether the most studied SNPs within *ABCB1* have an influence on treatment outcome in patients receiving GO based therapy in the randomized AAML0531 clinical trial. rs1045642, also commonly referred to as C3435T is a synonymous change (Ile1145Ile) in exon27 of *ABCB1* and has been shown to occur in partial LD with two coding SNPs rs1128503 (Gly412Gly) and rs2032582 (Ser893Ala/Thr). rs1045642-T allele has been shown to be associated with lower *ABCB1* expression in several studies^18–22^, and with higher expression in one study^23^. Hoffmeyer et al. linked TT genotype of rs1045642 with lower expression and activity of *ABCB1*^24^. Although more work is required to establish the clear relationship between *ABCB1* genotypes and calicheamicin efflux, our results are the first to indicate that presence of *ABCB1*- low-expressing rs1045642 TT genotype might increase intracellular abundance of calicheamicin due to reduced efflux in leukemic cells, which in turn can enhance chemo-sensitivity to GO. Our results warrant further investigation of *ABCB1* SNPs in context of gemtuzumab response in additional clinical cohorts.

Interestingly, SNP-SNP interaction between *ABCB1* SNP and recently reported CD33 splicing SNP implies that within CD33-rs12459419-CT, *ABCB1* genotype is critical for calicheamicin abundance and thus might contribute towards poor response observed within rs1045642-CT group. Our cohort was limited by sample size to perform SNP-SNP interaction evaluation, warranting the need for validation in additional cohorts.

Our results also open up opportunities to further investigate and validate impact of *ABCB1* SNPs in other GO randomized clinical trials. Once validated, *ABCB1* SNPs in conjunction with CD33-SNPs can provide more accurate prediction of response to GO opening up opportunities to personalize GO-therapy.

## Supplementary information

Supplementary Material
