# Supplementary material for: ABCB1 SNP predicts outcome in patients with acute myeloid leukemia treated with Gemtuzumab ozogamicin: a report from Children’s Oncology Group AAML0531 Trial
Source: Blood Cancer J. 2019 May 21;9(6):51. doi: 10.1038/s41408-019-0211-y (PMC6529443; doi:10.1038/s41408-019-0211-y)
Supplement: Supplementary file 1 — Supplementary Material [file 41408_2019_211_MOESM1_ESM.docx]

Supplementary Tables 1-4

| Supplementary Table 1. ABCB1 SNPs genotyped in patients from AAML0531 clinical trial. | | | | | | | |
| --- | --- | --- | --- | --- | --- | --- | --- |
| **SNP** | **Call Rate** | **Minor Allele** | **Major Allele** | **MAF** | **Gene** | **SNP Function** | **Amino acid change** |
| rs2235033 | 0.998 | C | T | 0.473 | ABCB1 | intron variant |  |
| rs1045642 | 0.999 | T | C | 0.465 | ABCB1 | synonymous codon | Ile1145Ile |
| rs1128503 | 0.998 | T | C | 0.432 | ABCB1 | synonymous codon | Gly412Gly |
| rs1922242 | 0.983 | T | A | 0.405 | ABCB1 | nearGene-5 |  |
| rs1922240 | 0.993 | C | T | 0.298 | ABCB1 | synonymous codon |  |
| rs1989830 | 0.993 | T | C | 0.288 | ABCB1 | UTR-3 |  |
| rs2235015 | 0.989 | T | G | 0.207 | ABCB1 | intron variant |  |
| rs2032582 | 0.998 | T/A | G | 0.415 | ABCB1 | missense | Ser893Ala/Thr |
| rs2235040 | 0.993 | A | G | 0.131 | ABCB1 | intron variant |  |
| **SNPs excluded** |  |  |  |  |  |  |  |
| rs3842 | **0.731** | G | A | 0.123 | ABCB1 | UTR-3 |  |
| rs9282564 | 0.994 | G | A | **0.007** | ABCB1 | missense | N21D |
| rs2229107 | 0.995 | A | T | **0.015** | ABCB1 | Near Gene-5 |  |
|  |  |  |  |  |  |  |  |
